# Research advances on gut microbiota dysbiosis and chronic liver diseases: a review

**DOI:** 10.3389/fmed.2026.1765047

**Published:** 2026-01-28

**Authors:** Guozhen Yang, Jiling Zhu, Minxin Wang, Sha She, Kai Dai

**Affiliations:** Department of Infectious Diseases, Renmin Hospital of Wuhan University, Wuhan, China

**Keywords:** alcoholic liver disease, chronic liver disease, cirrhosis, gut microbiota dysbiosis, gut-liver axis, viral hepatitis, metabolic-associated steatotic liver disease, mechanism

## Abstract

The gut microbiota is fundamental to human health, maintaining intricate symbiotic interactions with the host. Accumulating evidence highlights a critical association between gut microbiota dysbiosis and the initiation and progression of chronic liver diseases (CLDs). Particularly hepatitis B virus (HBV)/hepatitis C virus (HCV) infection, alcoholic liver disease (ALD), metabolic-associated steatotic liver disease (MASLD), and cirrhosis. This microbial imbalance may contribute to the progression of CLDs primarily via the “gut-liver axis,” the mechanisms involve gut barrier dysfunction, abnormal immune regulation, and metabolic alterations. This review synthesizes cutting-edge research on the interplay between gut dysregulation and CLDs, elaborating molecular mechanistic pathways including the TLR4/NF-κB signaling pathway, AMPK pathway, and farnesoid X receptor (FXR)-mediated bile acid signaling. Additionally, it discusses clinically oriented therapeutic strategies targeting microbiota modulation, including probiotics, fecal microbiota transplantation (FMT), and personalized dietary interventions, offering innovative insights for the prevention and management of chronic liver diseases.

## Introduction

The gut microbiota, a complex community of microorganisms residing in the gastrointestinal tract, plays a crucial role in maintaining human health. It is involved in essential functions such as digestion, metabolism, immune regulation, and protection against pathogens (1–3). The balance of this microbial community is vital for overall health, and its disruption, known as dysbiosis, is increasingly recognized as a contributing factor in various diseases, including chronic liver diseases (CLDs) (4). Research has shown a potential link between gut microbiota dysbiosis and the pathogenesis of CLDs. Dysbiosis can lead to increased intestinal permeability, allowing the translocation of microbial products such as lipopolysaccharides (LPS) into the bloodstream, which in turn triggers systemic inflammation and liver injury (5, 6). Furthermore, alterations in gut microbiota composition can affect liver metabolism, including lipid metabolism and bile acid synthesis, which may contribute to the progression of liver diseases (5, 7–9).

On the other hand, research suggests that the balance of the intestinal microbiota can be restored through dietary interventions, probiotic supplementation, and fecal microbiota transplantation, which in turn may alleviate chronic liver damage and reduce liver fibrosis. Therefore, it is essential to further investigate the underlying mechanisms linking intestinal microbiota dysbiosis to chronic liver diseases and to identify effective therapeutic strategies that can restore microbial equilibrium and support liver health. To ensure the comprehensiveness and rigor of this review, a systematic literature search was performed in PubMed, Embase, and Web of Science databases. Core keywords included “gut microbiota dysbiosis,” “gut-liver axis,” “chronic liver disease,” “HBV,” “HCV,” “alcoholic liver disease,” “MASLD,” “cirrhosis,” “probiotics,” “fecal microbiota transplantation,” “microbial metabolites,” and “mechanism.” Evidence priority was given to systematic reviews, Meta-analyses, and RCTs, followed by high-quality cohort studies and basic mechanistic research.

## Overview of gut microbiota

The gut microbiota, a complex and dynamic ecosystem, comprises trillions of microorganisms, including bacteria, viruses, fungi, and archaea, residing in the gastrointestinal tract (10–12). This microbiota plays a crucial role in maintaining host health by participating in various physiological processes, including digestion, metabolism, immune function, and the synthesis of essential vitamins and short-chain fatty acids (SCFAs) (13–16).

### Gut microbiota and dysbiosis

The gut microbiota consists predominantly of bacterial phyla such as Firmicutes, Bacteroidetes, Actinobacteria, and Proteobacteria, with each phylum containing various genera and species that perform specific functions (17–19). For example, Firmicutes includes beneficial species such as *Lactobacillus rhamnosus*, *Clostridium butyricum*, and *Faecalibacterium prausnitzii*; Bacteroidetes comprises Bacteroides fragilis and Bacteroides thetaiotaomicron, which are involved in polysaccharide fermentation; Actinobacteria includes Bifidobacterium infantis and Bifidobacterium longum; Proteobacteria includes potential pathogens such as *Escherichia coli* and *Salmonella enterica* (Table 1) (20). These microorganisms are involved in the fermentation of dietary fibers, which produces SCFAs like acetate, propionate, and butyrate, vital for colonic health and energy metabolism (21–23).

**TABLE 1:** Overview of core gut microbiota and their metabolites.

Microbiota phylum	Representative genera/species	Corresponding metabolites
Firmicutes	*Lactobacillus rhamnosus*, *Clostridium butyricum*, *Faecalibacterium prausnitzii*	Short-chain fatty acids (butyrate, propionate), lactic acid
Bacteroidetes	*Bacteroides fragilis*, *Bacteroides thetaiotaomicron*	Polysaccharide metabolites, secondary bile acids (deoxycholic acid)
Actinobacteria	*Bifidobacterium infantis*, *Bifidobacterium longum*	Acetate, B-group vitamins (B12, folate)
Proteobacteria	*Escherichia coli*, *Salmonella enterica*, *Klebsiella pneumoniae*	Lipopolysaccharides (LPS), ammonia, indole

Gut microbiota dysbiosis refers to an imbalance in the microbial community, often characterized by a decrease in microbial diversity and an overgrowth of pathogenic bacteria (13, 24, 25). Several factors contribute to dysbiosis, including antibiotic use, dietary changes, infections, and environmental exposures. For instance, the overuse of antibiotics can lead to a significant reduction in microbial diversity, allowing opportunistic pathogens to flourish and disrupt the gut barrier (26). Additionally, dietary patterns high in processed foods and low in fiber can alter the composition of gut microbiota, leading to metabolic disorders such as obesity and type 2 diabetes (27). Studies have shown that the imbalance of gut microbiota is closely linked to the occurrence, development, and prognosis of several liver diseases, including acute liver injury, viral hepatitis, cirrhosis, autoimmune liver disease, alcoholic liver disease (ALD), and metabolic-associated steatotic liver disease (MASLD) (28). This dysbiosis influences the degree of hepatic steatosis, inflammation, fibrosis, and even carcinogenesis through multiple interactions with the host immune system and other cell types, which is closely associated with the occurrence of chronic liver diseases (29). Understanding the mechanisms underlying dysbiosis is crucial for developing targeted interventions, such as probiotics and dietary modifications, to restore microbial balance and promote health (30–32).

## Gut microbiota dysbiosis and chronic liver diseases

**FIGURE 1:**
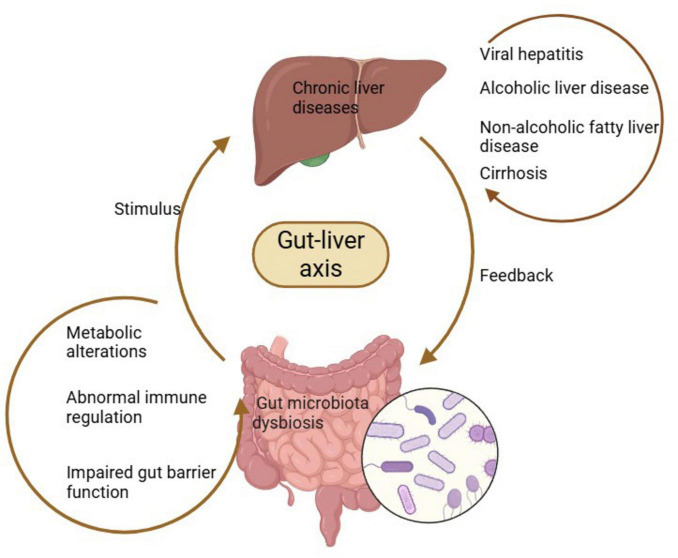
Mechanisms such as gut barrier dysfunction, abnormal immune regulation, and metabolic alterations can lead to gut microbiota dysbiosis, which is transmitted to the liver via the gut-liver axis, thereby promoting the initiation and progression of various chronic liver diseases (CLDs) including viral hepatitis (HBV/HCV infection), ALD, MASLD, and cirrhosis; conversely, the progression of CLDs further exacerbates gut microbiota dysbiosis through the same gut-liver axis, forming a persistent vicious cycle that drives continuous disease deterioration. Created with BioRender.com.

The gut microbiota plays a crucial role in maintaining human health and is increasingly recognized for its involvement in various diseases, including CLDs. Dysbiosis, a state of microbial imbalance, has been linked to the pathogenesis and progression of CLDs through several mechanisms. These mechanisms mainly include impaired gut barrier function, abnormal immune regulation, and metabolic alterations (Figure 1), which collectively contribute to increased susceptibility to infections and exacerbation of liver conditions (33, 34).

### Gut barrier dysfunction

Dysbiosis can lead to increased intestinal permeability, a condition often referred to as “leaky gut” (35). This phenomenon is mediated by downregulated expression of tight junction proteins, including zonula occludens-1 (ZO-1), Occludin, and Claudin-1, in intestinal epithelial cells. Pathogenic bacteria, notably *E. coli* and *Klebsiella pneumoniae*, secrete toxins such as cytolethal distending toxin (CDT) that induce degradation of tight junction proteins (36). Concomitantly, depletion of beneficial commensals (e.g., *L. rhamnosus* and *F. prausnitzii*) diminishes the biosynthesis of butyrate, which is an essential metabolite that sustains intestinal epithelial cell viability and preserves gut barrier integrity through activation of the AMPK signaling pathway (37).

Additionally, dysbiosis-induced inflammation downregulates the transcription of tight junction protein genes, further increasing intestinal permeability (38). This allows for the translocation of bacteria and their products, such as LPS, into the systemic circulation, which can provoke systemic inflammation and contribute to liver injury (5, 39). The disruption of the gut barrier is particularly concerning in patients with CLDs, as it can exacerbate the inflammatory response and further impair liver function. Studies have shown that alterations in gut microbiota composition, characterized by a decrease in beneficial bacteria and an increase in pathogenic strains, can lead to gut barrier dysfunction (38). This dysfunction not only facilitates the entry of harmful microbial products into the bloodstream but also contributes to the progression of liver diseases such as MASLD and ALD (40, 41).

### Abnormal immune regulation

The gut microbiota can mediate gut epithelial and immune cells interaction through vitamins synthesis or metabolic products. The microbiota plays a vital role in growth and development of the main components of human’s adaptive and innate immune system, while the immune system regulates host-microbe symbiosis. The gut microbiota is integral to the development and regulation of the immune system, and its disruption can lead to an imbalance between pro-inflammatory and anti-inflammatory responses (42). Mechanistically, dysbiosis-induced LPS translocation activates the TLR4/NF-κB signaling pathway in hepatic Kupffer cells and hepatic stellate cells, promoting the secretion of pro-inflammatory cytokines such as TNF-α, IL-6, and IL-1β, which drive hepatic inflammation and subsequent fibrogenesis (43, 44). Meanwhile, the reduction of butyrate impairs the differentiation of Tregs (regulatory T cells) in the gut and liver, while enhancing the activation of Th17 cells and their secretion of IL-17A—further amplifying the pro-inflammatory microenvironment (45).

Additionally, gut microbiota-derived metabolites such as indole (from Proteobacteria) can modulate the function of dendritic cells, leading to abnormal activation of adaptive (46). In chronic liver disease, this imbalance can result in immune dysregulation, characterized by an overactive inflammatory response that contributes to liver damage and increases the risk of infections. For instance, the presence of certain gut bacteria can stimulate the production of inflammatory cytokines, which further exacerbate liver inflammation and damage (38, 47). In other words, negative alteration in gut microbiota composition or gut dysbiosis, can disturb immune responses, which will make the host more susceptible to infections (48). Furthermore, dysbiosis has been associated with the activation of the gut-liver axis, where gut-derived signals influence hepatic immune responses, thereby enhancing the susceptibility to infections and liver disease progression (42, 48).

### Metabolic alterations

Dysbiosis also leads to significant metabolic changes that can increase the host’s susceptibility to infections and the progression of CLDs. As is well-known, The gut microbiota is responsible for the production of various metabolites, including SCFAs and bile acids, which play critical roles in maintaining gut health and regulating immune responses (5, 49). SCFAs, especially butyrate, primarily exert their effects via the AMPK signaling pathway: they suppress the expression of key hepatic lipogenic genes, including Srebp2, Fasn, Srebp-1c, Cd36, and Acc, to inhibit lipogenesis, while concomitantly activating fatty acid oxidation, thereby regulating lipid metabolism in hepatocytes and alleviating hepatic lipid accumulation (50, 51). Bile acids, as key signaling molecules, bind to FXR (farnesoid X receptor) in hepatocytes and intestinal epithelial cells to modulate glucose and lipid homeostasis, as well as regulate inflammation via the FXR-SHP pathway (52). Dysbiosis-induced reduction in SCFA production and alterations in bile acid composition (e.g., increased primary/secondary bile acid ratio) disrupt these metabolic pathways, leading to hepatic steatosis and metabolic dysfunction (53). Additionally, dysbiosis increases the production of microbial-derived ethanol and acetaldehyde, which directly induce hepatocyte injury in ALD and MASLD (54, 55).

Alterations in the composition of gut microbiota can disrupt the production of these metabolites, leading to an increased risk of metabolic disorders and liver disease (38, 47). For example, reduced levels of SCFAs due to dysbiosis have been linked to impaired gut barrier function and increased inflammation, both of which can exacerbate liver disease and increase susceptibility to infections (38). Moreover, changes in bile acid metabolism due to dysbiosis can further influence liver function and the risk of chronic liver disease (40). In summary, dysbiosis of gut microbiota significantly influences the pathogenesis of chronic liver diseases through mechanisms involving gut barrier dysfunction, abnormal immune regulation, and metabolic alterations (39, 47, 48). Understanding these mechanisms is essential for developing targeted therapeutic strategies aimed at restoring gut microbiota balance and improving liver health.

### Detection techniques and factors influencing microbiota research results

The inconsistency of gut microbiota research results in CLDs is partly attributed to differences in detection techniques and research factors. 16S rRNA sequencing can identify microbial taxa at the phylum and genus levels with high throughput but has limitations in species-level resolution (e.g., distinguishing *E. coli* from *E. fergusonii*) and functional annotation (56). By contrast, metagenomics sequencing research has accelerated the accumulation of genomic sequences of microbial species that had been inaccessible before. Analysis of the metagenomic sequencing data can reveal not only the species but also the functional composition of microbial communities, providing comprehensive information on microbial genes and functional pathways, enabling accurate species identification and prediction of metabolic potential, but this approach is constrained by high costs and computational complexity (57). Bioinformatics analysis pipelines also affect outcomes: QIIME2 is superior for 16S data analysis, while MetaPhlAn3 and HUMAnN3 are more suitable for taxonomic assignment and metagenomic functional annotation (58).

Additionally, geographic and dietary differences lead to variations in baseline gut microbiota composition (59)—Asian populations have higher abundance of Bacteroidetes due to high-fiber diets, while Western populations have higher Firmicutes. In cirrhotic patients, decompensated status is associated with more severe dysbiosis compared to compensated status (60). Studies have demonstrated that the abundance of Enterobacteriaceae, and Proteobacteria is increased in decompensated cirrhotic patients who eventually succumb to the disease (61). For HBV/HCV patients, antiviral therapy (e.g., entecavir for HBV, glecaprevir/pibrentasvir for HCV) can partially reverse gut microbiota dysbiosis by reducing hepatic inflammation, which should be considered when interpreting study results (62).

## Gut microbiota changes in chronic liver diseases

Chronic liver diseases are consistently associated with significant alterations in gut microbiota composition and function, a phenomenon that critically influences disease progression and clinical outcomes. Substantial heterogeneity exists in microbial dysbiosis patterns across distinct CLD etiologies—including viral hepatitis (HBV/HCV infection), MASLD, ALD, and cirrhosis—reflecting disease-specific host-microbiota interactions (Table 2). Key alterations encompass reduced microbial alpha-diversity, depletion of commensal taxa, and enrichment of pathobionts, collectively disrupting gut barrier integrity and promoting microbial translocation via the gut-liver axis (63). These perturbations drive hepatic inflammation, fibrogenesis, and metabolic dysregulation through mechanisms involving endotoxemia, bile acid metabolism impairment, and short-chain fatty acid depletion (64). A comprehensive understanding of etiology-specific microbial signatures is therefore essential for developing targeted therapeutic strategies aimed at restoring eubiosis, thereby mitigating liver injury and improving patient prognosis.

**TABLE 2:** Alterations in gut microbiota in different etiologies of chronic liver diseases.

Chronic liver disease subtype	Core microbiota changes	Key functional impacts	Associated pathological outcomes
Viral hepatitis (HBV/HCV)	Reduced microbial diversity; enriched Proteobacteria; depleted beneficial taxa (Firmicutes, Bacteroidetes); overgrown oral bacteria	Gut barrier disruption; microbial translocation; systemic inflammation; immune activation	Exacerbated hepatic inflammation/fibrosis; increased severe liver disease risk; progressive liver injury
Alcoholic liver disease (ALD)	Reduced microbial diversity; enriched pathogenic bacteria; depleted Lactobacillus/Bifidobacterium; alcohol-induced pro-inflammatory dysbiosis	Intestinal barrier impairment; endotoxemia; short-chain fatty acid depletion	Progression of alcoholic liver injury; hepatic inflammation/fibrosis
Metabolic dysfunction-associated steatotic liver disease (MASLD)	Shift to pro-inflammatory profile; enriched Firmicutes; depleted Bacteroidetes	Altered bile acid metabolism; LPS translocation; immune response modulation; metabolic dysregulation	Progression from simple steatosis to MASH/fibrosis; association with metabolic syndrome
Liver cirrhosis	Overgrowth of Enterobacteriaceae; depletion of protective commensals; reduced microbial alpha-diversity	Bacterial translocation; excessive ammonia production; gut barrier dysfunction	Spontaneous bacterial peritonitis (SBP); hepatic encephalopathy (HE); acute-on-chronic liver failure

### Gut microbiota changes in viral hepatitis (HBV/HVC infection)

Viral hepatitis, particularly caused by HBV and HCV, has been linked to significant dysbiosis in the gut microbiota (65). Studies have shown that patients with chronic HBV and HCV infections exhibit a decrease in microbial diversity, which is often accompanied by an increase in pathogenic bacteria such as Proteobacteria and a reduction in beneficial taxa like Firmicutes and Bacteroidetes (65–67). Confounding factors such as prolonged antibiotic use and PPI (proton pump inhibitor) administration can further alter the composition of gut microbiota, a phenomenon that is prevalent in viral hepatitis patients complicated with spontaneous bacterial peritonitis and gastroesophageal reflux disease. PPIs reduce gastric acid secretion, leading to the overgrowth of oral bacteria (e.g., *Streptococcus salivarius*) in the gut, which in turn exacerbates microbial translocation and hepatic inflammation (68, 69). Additionally, viral replication itself modulates gut microbiota by inducing systemic inflammation, which suppresses beneficial bacteria growth (70).

The gut microbiota’s composition can influence the severity of liver inflammation and fibrosis, as certain microbial profiles have been associated with increased liver damage and disease progression (71, 72). For instance, specific bacterial genera such as Escherichia and Shigella have been identified as potential markers for predicting the risk of developing severe liver disease in these patients (73, 74). Furthermore, the dysbiosis observed in viral hepatitis may exacerbate systemic inflammation, leading to further liver injury through mechanisms involving microbial translocation and immune activation (75). Thus, the interplay between gut microbiota and liver health in viral hepatitis underscores the potential for microbiome-targeted therapies to mitigate liver damage and improve clinical outcomes.

### Gut microbiota changes in MASLD and ALD

Metabolic liver diseases, such as MASLD, and ALD are characterized by distinct alterations in gut microbiota composition (76). In ALD, chronic alcohol consumption leads to dysbiosis, with a notable increase in pathogenic bacteria and a decrease in beneficial microbes. Phenomena such as antibiotic use and malnutrition also commonly occur in patients with ALD, and these confounding factors can exacerbate dysbiosis. Long-term antibiotic exposure significantly reduces microbial diversity, while a low-protein diet further impairs the growth of beneficial bacteria (e.g., Bifidobacterium) (54, 77). In addition, alcohol-induced intestinal epithelial injury directly alters the microbiota composition by creating a pro-inflammatory microenvironment that favors pathogenic bacteria (17, 78). Studies have reported a significant reduction in the abundance of *Lactobacillus* and *Bifidobacterium* species in patients with ALD, which correlates with increased intestinal permeability and systemic inflammation. Large amounts of endotoxins translocated from the gut strongly activate Toll-like receptor 4 in the liver and play an important role in the progression of ALD, especially in severe alcoholic liver injury (17, 54). Similarly, in MASLD, the gut microbiota shows a shift towards a more pro-inflammatory profile, with increased levels of Firmicutes and a decrease in Bacteroidetes, which is associated with the progression from simple steatosis to metabolic dysfunction-associated steatohepatitis (MASH) and fibrosis (79). In fact, gut dysbiosis is closely associated with hepatic steatosis and metabolic syndrome. The dysbiosis in these conditions is thought to contribute to the pathogenesis of liver disease through mechanisms such as altered bile acid metabolism, increased LPS translocation, and modulation of host immune responses (53, 80). Therefore, interventions aimed at restoring gut microbiota balance, such as dietary modifications, probiotics, and fecal microbiota transplantation, hold promise for improving metabolic liver disease outcomes.

### Gut microbiota changes in cirrhosis

Liver cirrhosis is one of the chronic liver diseases that can be complicated by episodes of decompensation such as variceal bleeding, hepatic encephalopathy (HE), ascites, and jaundice, with subsequent increased mortality. Infection remains one of the most frequent complications in patients with liver cirrhosis, with spontaneous bacterial peritonitis (SBP) being the most prevalent type. SBP is mainly induced by Gram-negative bacteria living in the intestinal tract, and translocating through the intestinal barrier, which in cirrhotic patients is defective and more permeable. Confounding factors such as long-term use of antibiotics (e.g., rifaximin), lactulose, and PPIs significantly affect the gut microbiota composition in patients with liver cirrhosis. For instance, rifaximin, which is used for the treatment of complications in cirrhotic patients, can markedly reduce the abundance of Enterobacteriaceae, but it may also lead to a decrease in beneficial bacteria (e.g., Lactobacillus); lactulose promotes the growth of *Bifidobacterium* and *Lactobacillus* through fermentation in the colon, thereby increasing the production of short-chain fatty acids (81, 82). Notably, decompensated cirrhosis is associated with more severe dysbiosis compared to compensated cirrhosis, including further reduction in microbial diversity and increased abundance of pathobionts (e.g., Enterobacteriaceae, Streptococcaceae) (60). Additionally, hepatic dysfunction itself also alters gut microbiota by reducing bile acid secretion, which is essential for maintaining microbial balance (40, 83). This dysbiosis is characterized by an overgrowth of pathogenic bacteria, including Enterobacteriaceae and a decrease in protective species, leading to increased susceptibility to infection (84, 85). The presence of SBP significantly worsens the clinical course of cirrhosis, contributing to acute-on-chronic liver failure and increased mortality rates (86). Mechanistically, the dysbiosis in cirrhotic patients not only facilitates bacterial translocation but also triggers systemic inflammation, exacerbating liver dysfunction and promoting further complications (60, 79). Consequently, strategies aimed at restoring gut microbiota balance, such as antibiotic prophylaxis and probiotics, are critical in managing the risk of infections in cirrhotic patients.

## Treatment of chronic liver diseases

The pivotal role of the gut-liver axis in CLDs has unveiled novel therapeutic avenues for their prevention and treatment. Specifically, interventions targeting the intestinal microbiome offer promising strategies to mitigate and repair hepatic injury while improving patient prognosis. Future research directions may emphasize the development of personalized therapeutic regimens incorporating dietary modifications, microbiota-targeted interventions, and advanced techniques including fecal microbiota transplantation (FMT), probiotics, and prebiotics (Table 3). This integrated approach seeks to reestablish intestinal microbial homeostasis, promote hepatic repair, and ultimately enhance the quality of life for patients with chronic liver diseases.

**TABLE 3:** Summary of microbiota-targeted therapeutic strategies for chronic liver diseases.

Therapeutic strategy	Core mechanisms	Key examples
Microbial therapies (probiotics/prebiotics/synbiotics)	Probiotics: replenish beneficial gut bacteria; strengthen intestinal barrier function; mitigate microbial imbalance	Bifidobacterium infantis; *Clostridium butyricum*; BL21
	Prebiotics: serve as metabolic substrates for beneficial bacteria; promote their proliferation/metabolic activity; generate SCFAs; improve gut barrier integrity	Lactulose; fructooligosaccharides
	Synbiotics: synergistically modulate gut microbiota composition/function; reduce blood ammonia and endotoxemia; alleviate systemic inflammation	Bifidobacterium longum+fructooligosaccharides
Fecal Microbiota Transplantation (FMT)	Restore intestinal microecological balance; enhance gut microbial diversity; strengthen intestinal barrier integrity; reduce infection recurrence risk	FMT for recurrent HE; FMT for recurrent CDI in liver disease patients
Dietary interventions	Modulate gut microbiota diversity/function; promote beneficial bacteria proliferation; reduce inflammatory response; regulate host metabolism/immune responses	MIND diet; high-fiber diet (fruits, vegetables, whole grains); caloric restriction+high-protein regimen for MASLD/cirrhosis
Personalized treatment strategies	Design targeted interventions based on individual characteristics (genetic profile, microbiota composition, disease phenotype); use AI/ML for multi-source data analysis to develop predictive models	Microbiota analysis-guided interventions (diet/probiotics/FMT); AI-assisted precise therapeutic regimen formulation

### Microbial therapies: probiotics, prebiotics, and synbiotics

Microbial therapy, encompassing probiotics, prebiotics, and synbiotics, represents a promising therapeutic approach to restore gut microbiota homeostasis and mitigate bacterial infection risk in patients with chronic liver disease (87).

Probiotics, as active beneficial microorganisms, mitigate microbial imbalances in liver diseases by replenishing beneficial gut bacteria and strengthening intestinal barrier function. A randomized controlled trial (RCT) involving 67 patients with HBV-related liver cirrhosis demonstrated that 12-week supplementation with Bifidobacterium infantis and Clostridium butyricum led to significant enrichment of dominant bacteria (Clostridium cluster I and Bifidobacterium) and marked reduction in Enterococcus and Enterobacteriaceae in the probiotic group. In addition, the indicators of intestinal mucosal barrier function were significantly improved after probiotic treatment, which may have contributed to the enhancement of cognitive function and the reduction of ammonia levels. The research demonstrates treatment with probiotics containing *C. butyricum* and *B. infantis* represents a new adjuvant therapy for the management of MHE in patients with HBV-induced cirrhosis (88). Probiotic application is associated with improvement in conditions such as minimal hepatic encephalopathy (MHE), highlighting their potential in the management of chronic liver diseases (66). Another RCT found that ALD mice diet with BL21 exhibited a significant reduction in hepatic oxidative stress, along with increased concentrations of superoxide dismutase, catalase and glutathione in the liver. Gut microbiota analysis revealed that BL21 intervention increased the relative abundance of Bifidobacterium and Akkermansia compared with the ALD group, this indicates that dietary supplementation with BL21 can ameliorate ALD by enhancing hepatic antioxidant capacity and modulating gut microbiota, and thus holds promise as a potential strategy for the prevention and treatment of this disease (89).

Prebiotics act as metabolic substrates for beneficial gut microbiota, enhancing their proliferation and metabolic activity. Their fermentation by intestinal commensals generates short-chain fatty acids (SCFAs) such as acetate, propionate, and butyrate—key metabolites that improve the gut barrier integrity and function, and modulate gut microbiota, glucose and lipid metabolism, as well as the inflammatory response and immune system, thereby contributing to gut-liver axis homeostasis and host metabolism (90). For instance, lactulose serves not only as a first-line treatment for HE but also functions as a prebiotic. A multi-center randomized controlled trial in China revealed that lactulose suppresses the growth of ammonia-producing bacteria such as *Streptococcus salivarius*, reduces ammonia production and absorption, and concurrently stimulates the proliferation of beneficial sugar-fermenting bacteria like *Bifidobacterium* and *Lactobacillus* (81, 91). Furthermore, numerous studies indicate that lactulose alleviates cognitive impairment in patients with chronic liver disease by inhibiting small intestinal bacterial overgrowth (SIBO) and bacterial translocation, as well as lowering serum levels of tumor necrosis factor-α (TNF-α), interleukins (ILs), and endotoxins (92).

Synbiotics, defined as combinations of probiotics and prebiotics, demonstrate a synergistic effect in modulating the composition and functionality of the intestinal microbiota. Clinical evidence indicates that synbiotic interventions significantly enhance liver function parameters, reduce systemic inflammation, and decrease infection incidence, particularly in patients with liver cirrhosis and hepatic encephalopathy (21). Patients with liver cirrhosis exhibit marked abnormalities in their intestinal microecosystem, characterized by the overgrowth of potentially pathogenic *E. coli* and *Staphylococcus* in feces. A clinical trial involving 97 consecutive cirrhotic patients with MHE but no overt hepatic encephalopathy demonstrated that synbiotic therapy significantly increased the fecal abundance of non-urease-producing Lactobacillus species at the expense of these other bacterial taxa. Modulation of the gut microbiota was associated with a significant reduction in blood ammonia levels and reversal of MHE in 50% of patients. Synbiotic therapy also led to a substantial reduction in endotoxemia. Improvements in the CTP (child-turcotte-pugh) functional class were observed in nearly 50% of cases (93). Furthermore, a synbiotic consisting of Bifidobacterium longum and fructooligosaccharides reduces blood ammonia levels and improves cognitive function in MHE patients (94). A systematic review shows that synbiotic supplementation reduces SIBO, promotes the proliferation of beneficial bacteria, decreases serum endotoxin levels, and alleviates hepatic encephalopathy symptoms in patients with liver cirrhosis (95). Moreover, the application of commensal bacteria such as Lactobacillus and Bifidobacterium effectively lowers blood ammonia and endotoxin levels and reduces the risk of MHE recurrence, which confers greater benefits on patients’ long-term prognosis. (95).

Microbial therapy not only demonstrates substantial benefits in the treatment of hepatic encephalopathy but also holds promise for intervening in metabolic liver diseases. A meta-analysis involving 11 RCTs and 741 patients with MASLD reveals that supplementation with probiotics, prebiotics, or synbiotics can enhance glucose homeostasis, reduce lipid levels, normalize liver enzyme profiles, and alleviate hepatic steatosis. Furthermore, the analysis indicates that in Caucasian populations, probiotics are more effective in improving MASLD compared to prebiotics or synbiotics administered in Asian or European populations (96). With advancing research on the gut-liver axis mechanism, integrating these microbiota-targeted therapeutic strategies into clinical practice is anticipated to offer novel approaches for promoting liver health and preventing infections.

### Fecal microbiota transplantation therapy

Fecal Microbiota Transplantation (FMT), as an emerging therapeutic strategy, has garnered increasing attention in the management of patients with chronic liver diseases complicated by recurrent infections. This therapeutic approach aims to restore intestinal microecological balance by transferring fecal material from healthy donors into recipients, thereby enhancing their overall health status (29). The transplanted fecal material comprises approximately 55% microbial communities and 24% soluble components, such as mucus, fat, protein, small molecules, and short-chain fatty acids (97). Compared with the targeted eradication of pathogenic bacteria through antibiotic therapy or the administration of specific probiotics, FMT enables the transplantation of a more comprehensive and stable intestinal microbiota system (98). In recent years, accumulating evidence has demonstrated that FMT can significantly reduce the risk of infection recurrence, particularly in cases of recurrent *Clostridioides difficile* infection (CDI), which is frequently observed in patients with liver disease who experience intestinal dysbiosis due to prolonged antibiotic use. Furthermore, FMT has demonstrated potential in ameliorating HE. As one of the most severe complications of liver cirrhosis, recurrent HE represents a significant cause of rehospitalization, neurological impairment, and mortality, often precipitated by upper gastrointestinal bleeding or severe infections. Although conventional antibiotic therapy can effectively control infections, it may further compromise the intestinal microbiota barrier and exacerbate HE progression. By restoring microbial diversity and enhancing intestinal barrier integrity, FMT offers a novel therapeutic strategy to address this challenge. Bajaj et al. (99) performed an open-label randomized clinical trial comparing the efficacy and safety of FMT from screened donors with standard of care (SOC) in patients with recurrent HE and liver cirrhosis. The results indicated that the FMT group not only exhibited significantly lower rates of adverse events and HE recurrence compared to the SOC group, but also demonstrated substantial improvements in cognitive function. Additionally, FMT conferred protective effects against hepatic necrosis and intestinal mucosal barrier injury.

The efficacy of FMT in these contexts demonstrates its potential as a therapeutic strategy for addressing complications associated with chronic liver disease. However, concerns surrounding safety and standardization persist. Adverse events, such as gastrointestinal discomfort and rare cases of multidrug-resistant organism transmission, underscore the need for stringent donor screening protocols. Addressing challenges in donor selection, regulatory oversight, and personalized approaches will be critical to optimizing FMT as a safe and effective therapeutic strategy (100). Additional investigations are required to optimize donor selection criteria, standardize procedural protocols, and evaluate long-term outcomes in order to fully harness the benefits of FMT within this patient population.

### Dietary interventions therapy

Dietary intervention represents a key strategy for modulating intestinal flora and reducing infection risk in patients with chronic liver diseases (101). Dietary composition significantly influences the diversity and functional capacity of gut microbiota, thereby regulating host metabolic processes and immune responses. Research indicates that diets abundant in dietary fiber (eggs, fruits, vegetables, and whole grains) promote the proliferation of beneficial bacteria, enhance SCFA production, and contribute to intestinal health maintenance (102). In patients with MASLD and cirrhosis, caloric restriction combined with high-protein regimens has been shown to increase microbial gene richness and improve clinical phenotypes (103). Conversely, high-fat and high-sugar dietary patterns tend to induce dysbiosis, characterized by reduced microbial diversity and heightened inflammatory responses. Findings from a cross-sectional analysis involving 450 adults diagnosed with MASLD revealed that greater adherence to the MIND diet was correlated with improved liver function, reduced systemic inflammation, enhanced metabolic health, as well as more favorable indicators of gut microbiome composition in MASLD patients. At the core of the MIND dietary pattern are polyphenol-rich foods (e.g., berries, leafy green vegetables), dietary fiber (whole grains, legumes), and unsaturated fats (nuts, olive oil) (104). Additionally, Clinical evidences demonstrate that targeted dietary interventions can substantially improve intestinal homeostasis, enhance hepatic function, and reduce infection incidence in chronic liver disease patients (105).

Notably, dietary interventions should be individualized based on disease severity: decompensated cirrhotic patients require moderate protein intake to avoid hyperammonemia, while MASLD patients benefit from low-fat, high-fiber diets with reduced added sugars. Future research should focus on identifying specific dietary patterns and components that effectively optimize gut microbial structure and function, thereby developing practical and effective strategies for infection prevention and health management in this patient population.

### Personalized treatment strategies

In the clinical management of chronic liver diseases, personalized treatment strategies are receiving increasing emphasis. This trend arises from a deepening understanding of the gut-liver axis and intestinal microbiota’s role in disease pathogenesis. Precision medicine focuses on designing targeted interventions based on individual patient characteristics, including genetic profile, microbiota composition, and disease phenotype, to enhance therapeutic outcomes and minimize adverse effects. For example, through microbiota analysis, clinicians can identify patient populations most likely to benefit from targeted dietary modifications, probiotic interventions, or fecal microbiota transplantation (106). Furthermore, the integration of artificial intelligence and machine learning algorithms for multi-source data analysis enables the development of predictive models, facilitating the formulation of more precise therapeutic regimens for chronic liver disease patients and further optimizing clinical outcomes (107). As research continues to elucidate the complexity of the gut microbiome and its intricate interactions with hepatic health, the implementation of personalized treatment strategies holds promise for substantially improving quality of life and long-term prognosis in patients with chronic liver diseases.

## Challenges and future perspectives

Despite significant progress in understanding the gut-microbiota-liver axis, several challenges remain in translating this knowledge into clinical practice. First, individual variability in gut microbiota: Gut microbial composition is influenced by genetics, diet, lifestyle, and disease status, leading to significant inter-individual differences. This variability makes it difficult to identify universal microbial signatures for CLDs, and may explain the inconsistent efficacy of microbiota-targeted therapies (108). Second, causal relationship vs correlation: Most studies have reported correlative associations between gut microbiota dysbiosis and CLDs, but the causal relationships remain unclear. Future studies using germ-free mice, microbial transplantation are needed to confirm the causal role of specific microbial taxa and metabolites. Third, lack of standardized detection methods: Current methods for gut microbiota analysis (16S rRNA sequencing, metagenomic sequencing) vary in accuracy and reproducibility, and there is no consensus on standardized sampling, processing, and analytical pipelines (56, 57). Fourth, limited long-term safety and efficacy data: Most clinical trials of microbiota-targeted therapies are small-scale and short-term, and long-term safety (e.g., risk of infection, immune disorders) and efficacy (e.g., prevention of disease progression to hepatocellular carcinoma) need to be evaluated in large-scale RCTs.

Future research directions should focus on the following aspects: (1) Precision medicine: Using multiomics technologies (metagenomics, transcriptomics, metabolomics, proteomics) to identify personalized microbial signatures and develop targeted therapies for different CLDs subtypes and patient populations. (2) Mechanistic exploration: Elucidating the molecular mechanisms of gut-microbiota-liver crosstalk at the single-cell and spatial levels, such as the interaction between specific microbial metabolites and hepatic immune cells or hematopoietic stem cells. (3) Novel therapeutic development: Developing next-generation probiotics (engineered bacteria producing therapeutic molecules), bacteriophages targeting pathogenic bacteria, and microbial metabolite analogs with enhanced stability and bioavailability. (4) Clinical translation: Establishing standardized gut microbiota detection platforms and therapeutic guidelines, and exploring the combination of microbiota-targeted therapies with traditional treatments (e.g., lipid lowering drugs, antiviral therapy) to improve clinical outcomes. (5) Prevention strategies: Identifying early microbial biomarkers for CLDs and developing dietary and lifestyle interventions to prevent gut microbiota dysbiosis and liver disease initiation.

## Conclusion

Dysbiosis of the gut microbiota and chronic liver diseases, particularly in the context of co-infection, represent a growing area of scientific inquiry. This review seeks to elucidate the intricate relationship between intestinal microbial composition and the progression of chronic liver diseases (HBV/HCV infection, MASLD, and cirrhosis), which holds significant implications for developing novel diagnostic and therapeutic approaches while improving patient outcomes. Gut microbiota dysbiosis can exacerbate hepatic inflammation, fibrosis, and overall disease advancement. The gut-liver axis serves as a critical pathway, mediating hepatic dysfunction and disease severity through microbial metabolites and inflammatory signaling (e.g., TLR4/NF-κB, AMPK pathways). This mechanistic understanding not only deepens insights into the pathogenesis of chronic liver diseases but also identifies promising intervention targets. Furthermore, the gut microbiota has emerged as a compelling therapeutic focus, with interventions including probiotic and prebiotic supplementation, alongside dietary modifications, and FMT, demonstrating potential for restoring microbial homeostasis and promoting hepatic health. However, a key challenge remains in optimizing intervention strategies to enable precise applications tailored to individual microbiota profiles and clinical presentations. The advancement of personalized medicine in this domain necessitates rigorous clinical investigations to establish the efficacy and safety profiles of various interventions. The integration of advanced technologies such as metagenomics and metabolomics will further elucidate the microbiome’s role in liver pathologies and provide a foundation for refining therapeutic strategies. In terms of treatment safety, microbiota-targeted therapies carry certain risks that require clinical attention. Probiotics are generally safe, but in cirrhotic patients with immunodeficiency, the risk of sepsis should be vigilantly monitored. FMT has the potential risk of transmitting infectious diseases and inducing adverse reactions, which can be mitigated through rigorous donor screening and standardized procedures. Dietary interventions should avoid extreme diets (e.g., excessive protein intake in patients with decompensated cirrhosis may trigger hepatic encephalopathy), and the dosage should be adjusted based on the patient’s tolerance and nutritional status. Prior to initiating microbiota-targeted therapy, clinicians should conduct an individualized risk-benefit assessment, closely monitor patients for adverse events during treatment, and administer timely symptomatic management.

In conclusion, the intimate relationship between intestinal microbiota dysbiosis and chronic liver diseases, particularly under infectious conditions, establishes a promising avenue for future scientific exploration. Recognizing the central role of intestinal microecological regulation in therapeutic approaches will facilitate the development of innovative strategies that not only ameliorate hepatic health but also significantly enhance patients’ overall quality of life.
